# Inroads to Predict *in Vivo* Toxicology—An Introduction to the eTOX Project

**DOI:** 10.3390/ijms13033820

**Published:** 2012-03-21

**Authors:** Katharine Briggs, Montserrat Cases, David J. Heard, Manuel Pastor, François Pognan, Ferran Sanz, Christof H. Schwab, Thomas Steger-Hartmann, Andreas Sutter, David K. Watson, Jörg D. Wichard

**Affiliations:** 1 Lhasa Ltd., 22-23 Blenheim Terrace, Woodhouse Lane, Leeds, LS2 9HD, UK; E-Mails: Katharine.Briggs@lhasalimited.org (K.B.); David.Watson@lhasalimited.org (D.K.W.); 2 Research Programme on Biomedical Informatics (GRIB), Fundació IMIM, Universitat Pompeu Fabra, PRBB, Dr. Aiguader 88, 08003 Barcelona, Spain; E-Mails: mcases@imim.es (M.C.); manuel.pastor@upf.edu (M.P.); fsanz@imim.es (F.S.); 3 Department of Preclinical Safety, Novartis Institutes for Biomedical Research (NIBR), Postfach CH-4002, Basel, Switzerland; E-Mail: david.heard@novartis.com; 4 Molecular Networks GmbH, IZMP, Henkestr. 91, 91052 Erlangen, Germany; E-Mail: schwab@molecular-networks.com; 5 Bayer HealthCare, Investigational Toxicology, Müllerstr. 178, 13353 Berlin, Germany; E-Mails: thomas.steger-hartmann@bayer.com (T.S.-H.); andreas.sutter@bayer.com (A.S.); joerg.wichard@bayer.com (J.D.W.)

**Keywords:** predictive toxicology, *in silico* toxicity, *in vitro* toxicity, *in vivo* toxicity, Knowledge Management, Expert Systems, Decision Support System, Data Integration, Manual Curation, ontology, histopathology, computational models, QSAR, data sharing

## Abstract

There is a widespread awareness that the wealth of preclinical toxicity data that the pharmaceutical industry has generated in recent decades is not exploited as efficiently as it could be. Enhanced data availability for compound comparison (“read-across”), or for data mining to build predictive tools, should lead to a more efficient drug development process and contribute to the reduction of animal use (3Rs principle). In order to achieve these goals, a consortium approach, grouping numbers of relevant partners, is required. The eTOX (“electronic toxicity”) consortium represents such a project and is a public-private partnership within the framework of the European Innovative Medicines Initiative (IMI). The project aims at the development of *in silico* prediction systems for organ and *in vivo* toxicity. The backbone of the project will be a database consisting of preclinical toxicity data for drug compounds or candidates extracted from previously unpublished, legacy reports from thirteen European and European operation-based pharmaceutical companies. The database will be enhanced by incorporation of publically available, high quality toxicology data. Seven academic institutes and five small-to-medium size enterprises (SMEs) contribute with their expertise in data gathering, database curation, data mining, chemoinformatics and predictive systems development. The outcome of the project will be a predictive system contributing to early potential hazard identification and risk assessment during the drug development process. The concept and strategy of the eTOX project is described here, together with current achievements and future deliverables.

## 1. Introduction: Shortcomings of Toxicology in Current Drug Development

The main barrier for a new drug to enter into clinical development is the preclinical evaluation of toxicity, where the systemic rodent and non-rodent toxicity studies are the pivotal investigation paradigms (as described in various guidelines e.g. International Conference of Harmonisation Topic M 3 (R2)) [1]. Approximately 35% of all drug development projects fail as a result of toxicity detected during preclinical safety studies [2], therefore animal studies are an important safeguard for the safety of patients. In order to front-load identification of toxic effects into earlier phases of development, where several candidates are under investigation and lead compounds can still be modified, *in vitro* screening assays have been developed for a variety of toxicological endpoints (for an overview see [3]). While predictive screening assays are useful for endpoints such as genotoxicity and hERG inhibition, the complex interplay of factors that lead to systemic or organ toxicity *in vivo* is not effectively represented *in vitro*. Assays for phospholipidosis [4], off-target pharmacology profiling and inhibition of the hERG channel [5] *in vitro* are useful to identify hazards and thus contribute to the design of more hypothesis-driven *in vivo* studies. However, while *in vitro* approaches can offer a certain mechanistic insight, they are rarely able to provide risk assessment information for the *in vivo* situation, *i.e.*, a decision to terminate a compound or a chemical series is rarely based on the outcome of such assays.

In terms of computational approaches, there are *in silico* models focusing on, for example, the prediction of genotoxicity, skin sensitization and hERG inhibition, that show a reasonable predictive accuracy [6–8]. Nevertheless, the available computational models to predict *in vivo* toxicity in general and organ toxicity in particular [9–12] typically cover only a narrow chemical space due to the small training sets available, and are of poor predictive value, thereby limiting their use in drug discovery projects. General issues with computational modeling of *in vivo* toxicity arise from the complexity of the endpoints, the need to implement a prediction of exposure for risk assessment and the lack of data sets with appropriate size, quality, and coverage of the large chemical space in which the prediction must be made (a prerequisite for building robust models). Indeed, most *in silico* models have been developed for the prediction of simpler endpoints represented by binding to a single biomolecule, and their extension to *in vivo* endpoints will probably require the prediction of a variety of involved mechanisms or pathways and their subsequent integration using methods that simulate theunderlying physiology.

As a result of the lack of reliable *in silico* and *in vitro* models for the prediction of *in vivo* toxicity, most pharmaceutical companies have introduced an early *in vivo* ‘minitox’ assay with repeated dosing over one or two weeks in rodents and/or non-rodents, in order to predict the outcome of the larger, more expensive GLP (Good Laboratory Practice) studies. The pervasiveness of this practice clearly demonstrates that there is still a need for better and earlier toxicity prediction. Improved *in silico* models could help to optimize the design of such systemic toxicity studies, or even replace some of them, thus contributing to the replacement, refinement and reduction of animal use in research and testing, known as the principle of the 3Rs [13].

Since the definitive introduction of the good laboratory practice (GLP) principles in 1981, all preclinical toxicity studies generated by the pharmaceutical industry strictly follow GLP rules, ensuring high data quality in the study reports. Whereas every company archives all generated data in a fully traceable manner, this information is not stored in a way that allows retrieval of study conclusion data in a structured format for the generation of simple statistics across the reports of a given company, let alone the entire industry. Indeed, it would be of great interest to the industry to be able to analyze this data and learn how to avoid costly failures in the future. The data in the collected preclinical toxicity reports of pharmaceutical companies represents the most important data source for improved *in silico* toxicity model building. Perhaps surprisingly, none of the 13 companies involved in eTOX project currently has the ability to answer simple questions from their own data such as: “What type of compound-induced liver toxicity is the most commonly observed in rat across all studies?” or “What is the translatability of toxicity findings across species?”. Such questions could be answered by extracting the data from study reports and putting this information into a structured database. This is especially true if all companies could share this data. Of course, many more complex and meaningful questions could be generated to exploit this toxicological “gold mine” and more may be learnt from the past to improve future medicines. Currently, very few pharmaceutical companies have unilaterally decided to reorganize their preclinical toxicity archives and extract the data to build searchable and mineable databases given the considerable investment in terms of time and money that is required. Hence, one of the main goals of eTOX was to also organize the extracted data into a searchable structure with appropriate tools.

Since eTOX is still in the phase dominated by data extraction, collection and database construction, the achievements with regard to modeling and predictive tools are still limited. Therefore, the main objective of this article is to provide an overview on the concept and strategy of the eTOX project to a broader audience. In addition, the design of the database and the strategy to overcome the problems of data sharing in the context of intellectual property will be explained. Although the early predictive models will be reported, subsequent publications will focus on details of the modeling approaches and performance of the predictive tools developed in the frame of eTOX.

## 2. The Innovative Medicines Initiative as Framework For Improving Toxicity Assessment in Drug Development

In 2008, a group of preclinical safety scientists from the pharmaceutical industry recognized that, collectively, they were sitting on the largest collection of unpublished, high quality *in vivo* toxicity data in existence. More importantly, they realized the potential benefit of a collaborative approach to the sharing of corporate toxicology data that could provide a significant advance in predictive toxicology by overcoming some of the shortcomings described above. Toxicity data acquired during drug development is not routinely published or shared in public databases owing to the confidential nature of the research that generates the data. However, sharing these data would not only allow the comparison of new structures to already existing data and thus contribute to the principle of the 3Rs by a more refined design of *in vivo* studies, but would also constitute the basis for the development of more reliable computer models to assist in the prediction of *in vivo* toxicity.

As a consequence, the European Innovative Medicines Initiative (IMI), a public-private partnership of the European Union and the European Federation of Pharmaceutical Industries and Associations (EFPIA), launched a call for a project to be funded to achieve this goal of data sharing and building of new *in silico* safety models. IMI [14] plays an important role in this endeavor, being Europe's largest public-private initiative aiming to speed up the development of better and safer medicines for patients. IMI supports collaborative research projects and builds networks of industrial, small and medium enterprises (SMEs) and academic experts in order to boost pharmaceutical innovation in Europe.

Eleven expressions of interest from consortia of academic institutions and small-to-medium enterprises (SMEs) were submitted for the above-mentioned topic and subsequently evaluated by independent experts during 2008. The project selected was “Integrating bioinformatics and chemoinformatics approaches for the development of expert systems allowing the *in silico* prediction of toxicities” (eTOX, “electronic toxicity” [15]) submitted by the academic institutions and SMEs listed in Table 1. The main objectives of this project are: to identify and implement ways for data sharing while safeguarding intellectual property; to build a harmonized toxicological database; and to use this database for the development of predictive models. In total, thirteen EFPIA companies have decided to participate in the project consortium (see Table 1).

Proprietary structural and pharmacological knowledge on chemical entities represent the main assets of each pharmaceutical company. It was therefore crucial to identify during the preparatory phase of the project, ways to share this knowledge without endangering a company’s competitive advantage. After overcoming this initial hurdle, the eTOX project officially started on 1 January 2010.

## 3. Improving Toxicity Prediction—The eTOX Project

**Figure 1:**
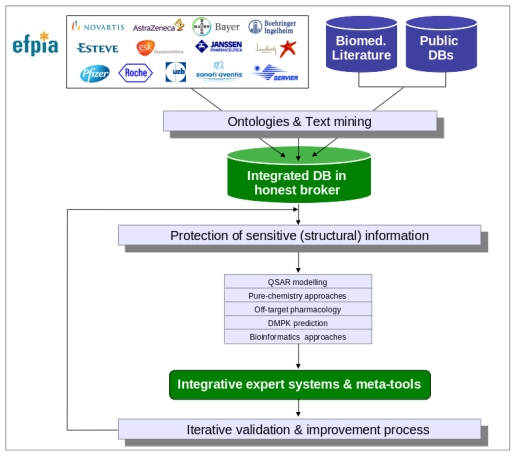
Overview of the eTOX project strategy. eTOX collects toxicological data from pharmaceutical (EFPIA) companies and public sources, and incorporates them into a database hosted by the “honest broker” to safeguard IP issues related to these data. The database will then serve as a source for the development of *in silico* models to predict the *in vivo* toxicity of new drugs.

The eTOX project is focused on the development of innovative strategies for the *in silico* prediction of the *in vivo* toxicities of drugs, and their implementation into integrated and customizable software tools. The intended predictive system, eTOXsys (see section 3.7), aims to significantly improve the quality of the current state-of-the-art computational predictions [16,17] for the pharmaco-toxicological profiles of new drug candidates. The eTOX project has some aspects in common with other European projects and initiatives such as, OpenTox (focused on environmental toxicity) [18] or the OSIRIS project [19]. However, eTOX is substantially different in terms of the concept, strategy and scientific and technological approaches applied as described below (see Figure 1 for an overview of the eTOX project strategy):

- Creation and maintenance of an integrated database of high-quality data of *in vivo* preclinical toxicology and *in vitro* pharmacology for drug-like molecules [20]. The selection and classification of the records to be shared among the EFPIA companies, the development and application of appropriate standards and ontologies (with a focus on histopathology descriptions), and setting up of strategies for data quality assessment, will be key tasks for the establishment of the intended, integrated database. This database will be hosted by one of the partners (Lhasa Limited), acting as “honest broker” of the data.
- Development and implementation of strategies (procedural and technological) for protecting sensitive information coming from the participating pharmaceutical companies. Since part of the molecular structures will be particularly sensitive information to be protected, encoding by means of the irreversible transformation of structures into molecular descriptors is to be considered [21].
- Development and application of text mining techniques required for the automatic exploitation of biomedical literature and legacy reports of the pharmaceutical industry. The intended text mining techniques will go beyond the classical co-occurrence analysis by incorporating natural language processing [22].
- Application of computational techniques for the prediction of pure chemistry-related toxicology (e.g. cationic amphiphilic drugs and phospholipidosis) by means of sub-structure or fragment-based approaches, as well as by the detection of toxicophores.
- Implementation of strategies for the prediction of off-target pharmacology by means of the automatic analysis of similarities between the studied compounds and extensive collections of biologically annotated ligands stored in chemogenomic databases, as well as by performing docking simulations of the studied compounds in structural models of key off-target interaction such as the hERG K+ channels [23].
- *In silico* prediction of the interaction of drugs with a relevant panel of drug transporters [24] as well as metabolism predictions will be developed, since drug transport and metabolism play a key role in triggering or avoiding some of the toxic or side effects of drugs [25]. Aspects of metabolism will be covered using the Meteor software (Lhasa Limited, Leeds, UK), also see Marchant *et al.* [26], MetaSite (Molecular Discovery Ltd., Pinner, UK), also see Cruciani *et al.* [27] and CRAFT (Molecular Networks GmbH, Erlangen, Germany) programs.
- Development of large-scale QSAR models for the prediction of toxicity outcomes. Molecular and physicochemical descriptors and multivariate analysis techniques, hierarchical and block-oriented methods will be applied, as well as neural networks and Bayesian methods [28].
- Incorporation of -omics data and cross-omics mapping in order to understand and model toxicological phenomena observed *in vivo* [29]. The modeling of biological pathways in a way that allows for the assessment of the perturbations produced by drugs is one of the aims of this project. Comparative genomics analysis will also be implemented to address the variations in the toxicological features observed in different species.

**Figure 2:**
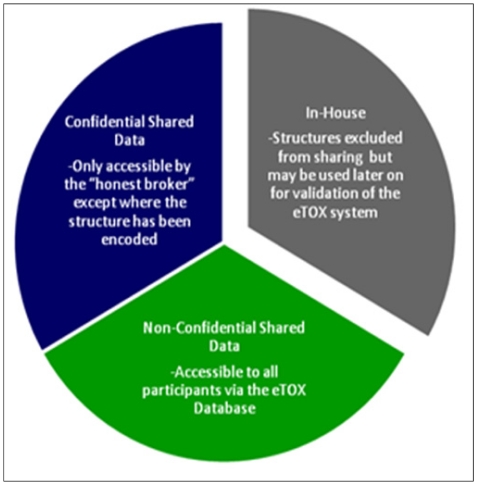
The sensitivity classifications used in the eTOX project

As each of the aforementioned approaches on their own will generate a prediction that forms part of a bigger picture, or prediction, for the chemical, a key activity of the project will be the development of integrative algorithms (including decision trees, reasoning and consensus models), together with expert systems reasoning (*i.e.*, Derek Nexus [30]), in order to combine the series of particular predictions into a more powerful and comprehensive strategy and software framework for toxicological assessment. However, in contrast to projects that are open to the public, the eTOX project contains a large amount of proprietary data donated by the consortium members which requires a level of confidentiality with respect to chemical structures and targets (see Figure 2). This requires a specific effort to protect sensitive information that is not of concern to a web based open information system as that provided by OpenTox. The eTOXsys will use web services not exposed to the public. In the final version, the system will be installed behind corporate firewalls, protecting the confidentiality of compounds under investigation.

An iterative process consisting of system development, experimental validation, critical assessment and system improvement will be devised. The experimental validation rounds will be carried out on series of compounds not used for the development of the applications. The characterization, evaluation and validation of QSARs will be performed following the OECD principles [31], taking advantage of the participation of one of the eTOX partners in the OECD Expert Group.

### 3.1. Construction of the eTOX Database

The eTOX consortium is currently building a large database containing proprietary compound and animal safety data from pharmaceutical companies, previously inaccessible to anyone but the respective owners. This will be integrated with publicly available data sources. As mentioned above, the database will be hosted by Lhasa Limited, which acts as “honest broker”. Lhasa Limited was selected on the basis of their previous experience in data sharing projects and the fact that Lhasa had already developed a searchable toxicological database that could be used and modified for the purposes of eTOX. Lhasa Limited is a not-for-profit, charitable organization that exists to promote the sharing of data and knowledge in chemistry and life sciences. This organization has extensive experience in the role of honest broker for the sharing of mutagenicity data on impurities stemming from the drug manufacturing process and the results of repeated dose studies on pharmaceutical excipients [26]. Lhasa Limited has previously developed the Vitic Nexus software, a chemically intelligent toxicity database, to facilitate such sharing.

The schema of this database is self-describing and can easily be modified to meet the specific requirements of individuals or groups. Vitic Nexus incorporates editing tools to enable in-house data to be imported and edited, together with an SDFile import utility for uploading new data in batch mode. The software supports similarity as well as exact match and substructure searching. Sophisticated searches can be built up by defining multiple criteria and combining them using Boolean logic. In this way, chemical structure searches can be combined with text searches, including toxicological criteria and experimental protocol constraints. Multiple databases can be searched simultaneously and the results from all searched databases displayed together.

The establishment of data-sharing strategies requires agreement on the format and extent of data to be contributed by each participating organization. The primary focus for data collection are the systemic toxicity studies (1–4 week repeated dose studies in rodent, dog and other non-rodent) but data collection is intended to be extended to longer studies, as well as to pharmacokinetic/toxicokinetic studies, *in vivo* safety pharmacology and gene expression data sets during the course of the project. Of the several thousand systemic toxicity studies already identified across all the participating EFPIA companies, most are rat studies. In addition, approximately 925 new repeat dose toxicity studies are performed in total each year within the participating EFPIA partners, of which about 550 are rat studies. It is hoped that the majority of these studies will also be made available to the project.

The schema for the database has been developed in an iterative manner. The first draft, created by Bayer Healthcare, was implemented into Vitic Nexus by Lhasa Limited and several improvements were made during the course of a pilot study, based on the feedback received from participating contract research organizations (CROs) extracting the data together with the EFPIA partners.

A further review of the schema is planned when the database is more fully populated. As a next step, schemas covering non-rodent species and further types of preclinical studies such as receptor and enzyme screening data will be implemented. Additional tables, if needed, will be defined, based on examples of the data to be captured. Subgroups will be set up consisting of consortium members with experience of the data being captured who will advise on:

- Those values/fields users are likely to include in their queries;
- Formats for displaying or reporting the results;
- Those values/fields important for determining data quality;
- Those values/fields important for developing predictive models.

### 3.2. Safeguarding Intellectual Property

The development and implementation of procedural and technological strategies for the protection of sensitive information is required in order to allow sharing of information among the participating pharmaceutical companies. A combination of legal contracts, physical access controls, software controls, sensitivity levels (Figure 2) and structure masking were elaborated to ensure the optimal protection of the sensitive, shared data. Obtaining permission to release data from EFPIA legacy reports to the consortium represents a crucial and potentially time-limiting step for the eTOX project. The procedures for getting such permissions vary between companies: compounds which have fallen into the public domain may be considered as the least critical and, whilst some companies regard data on marketed substances as most sensitive, for others it is data from development projects. Structures from terminated projects may gain importance, as they may be ‘re-discovered’ for different projects or indications. Most companies need to get permission for each structure and/or for each report individually from their R&D heads and their patents & licensing groups. Bayer Healthcare has elaborated a procedure to get a general permission for full or restricted sharing. Classification of structures or reports according to confidentiality levels is being run in parallel to data extraction in order to speed progress towards the final goal, the donation of data to the eTOX database.

Data classified as “non-confidential shared data” will be accessible to all project participants. Data classified as “confidential shared data” will be held by the honest broker, but will be accessible only by the original owner of the data. Modelers who intend to mine these data will have to agree a secrecy agreement with the data owner. The honest broker will not only provide the physical barrier system but also manage the secrecy agreements and control subsequent data access. In addition, all partners have signed an agreement to not attempt to reverse engineer masked structural information that they will obtain during the project.

### 3.3. Data Extraction and Gathering

Extracting and gathering the data from legacy reports represents the rate-limiting step for all subsequent data mining and predictive model building. The process was initiated in parallel with the development of the database and will continue throughout the duration of the project. In line with the priorities set in the project proposal, data collection commenced with summary data from systemic toxicity studies in rodents. It is planned to incorporate raw data into the final database wherever possible in order to be able to mine for previously unknown relationships and on an individual animal basis, if needed. A survey of the EFPIA partner companies at the start of the project estimated the number of repeat dose toxicity reports (1 to 4 weeks administration) eligible for the project to be roughly 10,736, comprising around 1,900 different chemical structures. The current evaluation of the existing reports and the accompanying intellectual property situation suggests that approximately 20% of non-confidential data sets can be fully shared among the partners.

In order to make the data available in a machine-readable format, a data extraction process is required. Manual data extraction inside the EFPIA companies was previously proven to be both time and cost-intensive and was identified as a potential, major bottleneck of the project.

**Figure 3:**
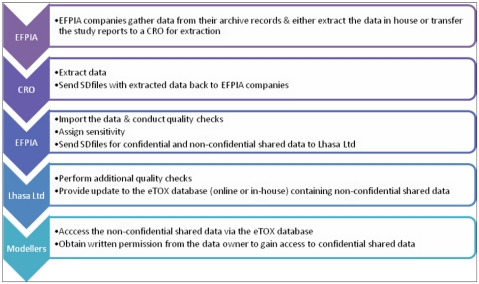
Summary of the data transfer process.

Several subcontractors offering manual or semi-automated data extraction were evaluated in a feasibility study using a small number of reports with diverse characteristics. The aim of this ‘data extraction pilot study’ was to identify contract research organizations (CROs) with sufficient capacity, good quality standards, assurance of confidentiality and competitive offers with respect to time and costs. Following the evaluation, the EFPIA partners agreed a shortlist of three CROs and it will be at the discretion of each EFPIA partner to decide whether they will enter the data themselves or employ one of the shortlisted CROs to do the data extraction for them. The data extraction process is briefly outlined in Figure 3. Currently, there are 2091 reports identified for sharing within the project, of which 1648 have been submitted to CRO’s for extraction. Of these, 106 of which have been already completed and 15 are in the Vitic database. It is expected that the majority of identified reports will be accessible to the modelers in the project by the end of this year.

**Figure 4:**
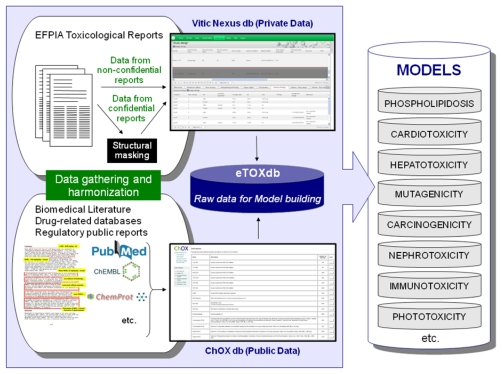
Gathering and harmonization of data from EFPIA partners and public sources: Strategy to populate the eTOXdb with data suitable for model building.

Efforts are underway to gather relevant data from public sources (e.g. scientific literature and non-proprietary databases, see Figure 4). Published datasets that are thought to be of good quality and appropriate for the project as training and validation datasets for predictive models have been identified and made available in a repository within the eTOX intranet. This repository, monthly updated and maintained by Fundació Institut Mar d’Investigacions Mèdiques (FIMIM), will be gradually included into the ChOX database. ChOX [32] is an internal repository based on the ChEMBL [33] database implementation, developed by European Molecular Biology Laboratory (EMBL). The first version contains 2D structural and physicochemical information on 153,520 distinct compounds annotated with bioactivity data on 384 proteins (particularly populated with CYP450, transporters and off-target data with relevance to toxicity assessment). The biological information that is currently incorporated corresponds to 415,051 bioactivity data points across seven species, extracted from 9,101 publications.

The considered bioactivities are essentially binding data (IC_50_ and Ki) and pharmacological data (EC_50_). The pharmacokinetic (PK) data so far included in ChOX is for bioavailability, clearance, volume of distribution and half-life, and includes data from four species. There are several ways to browse and to analyze the data, including exact, substructure and similarity structural searches, specific physicochemical parameters (MW, AlogP, PSA), type of activity (IC_50_, K_i_, EC_50_) and molecular target.

Future plans include the addition of publicly available data from toxicogenomics experiments via array express or GEO (Gene Expression Omnibus) [34] with links provided from the ChOX interface. The user will also have the ability to query ChemProt and ChEMBL in parallel to ChOX.

### 3.4. Development of Database Standards

The pilot data extraction study highlighted the need for a consensus to be reached on how the database schema should be populated. Therefore, the questions and answers generated during the pilot study have been used to define data entry guidelines to ensure consistency among the various CROs and EFPIA partners entering data. For modeling purposes, it is important to enter both positive and negative findings and to include data for control groups. Any evidence of background findings such as, for example, cardiomyopathy will also be captured.

The EFPIA partners and Lhasa Limited will carry out quality assessments and consistency checks on the extracted data. Owing to the heterogeneity of the study data, it has been necessary to identify ways to harmonize the measurement units used for quantitative data and to develop ontologies.

### 3.5. Ontologies for Preclinical Safety

Ontologies are formal representations of knowledge within a specific domain that show the relationships between different concepts in that domain. Ontologies and controlled vocabularies with synonym mapping are extremely important for a project like eTOX because they allow the terms that have evolved over time, across all the different companies, and those in public literature to be mapped to a single preferred term. As a result of their hierarchical structure, ontologies allow the grouping of findings from different studies at different levels of the tree, which can help solve the issue of different pathology descriptions of a finding with different levels of specification (e.g. “chronic inflammation” *vs.* “inflammation”). This is essential for cross study data analysis as well as the development of models. It is hoped that this work will also contribute to an industry standard ontology for preclinical findings.

In previous efforts to create microscopic finding ontologies, a finding was always linked with an organ, *i.e.*, “Liver necrosis” and treated as a single term. This means that the term “necrosis” must be entered many times into the ontology as it can occur in different tissues. In eTOX, it was decided to separate the finding from the anatomy so that the term necrosis stands alone and the term liver stands alone. Based on previous experience accumulated in Novartis, it was decided to follow a different approach for developing a preclinical safety ontology, taking into account the following requirements: The ontology must,

- Be easy to maintain.
- Allow a flexible mapping of findings for later computer modeling. For the example of liver necrosis when using a machine learning approach, the feature “finding” (e.g. necrosis) will be treated separately from the feature “anatomical region” or “organ” (e.g. liver) which will allow the machine learning algorithm to automatically analyze compounds causing necrosis across all tissues or all findings in liver.
- Enable the creation of hard links (where needed) between the different ontologies (i.e. to link the pathology finding “hyperostosis” to the rather high level anatomy term “bone”).

While the ontologies are important for later modeling, they are not required for initial data capture from study reports. Most of the EFPIA companies do not have access to the lists of terms used in their company until the verbatim terms are captured from the reports. Therefore, it was decided to extract verbatim, findings terminology from the reports and to map them into the ontologies afterwards. The listing of ontologies/vocabularies that need to be created and some key characteristics are described below:

- *The Anatomy Ontology*: A list of terms and relationships that describe the anatomical locations, organs or tissues from any animal used in preclinical safety experiments. Starting with the Adult Mouse Anatomy Ontology created at the Jackson Laboratories [35], all the terms in use in the Novartis preclinical databases have been mapped to this ontology as synonyms and expanded with new terms where appropriate. This is a relatively complete ontology and it was found that of the 1,600 terms from all species existing in the Novartis database, almost 90% were mapped as synonyms and only about 150 had to be added as new preferred terms. While the anatomy ontology is based on a mouse ontology, it is useful for all species used in preclinical safety studies with only minor modifications, such as the creation of links to a particular species for some terms (i.e. linking “harderian gland” to “rodents” in the species ontology).
- *The Microscopic Pathology Ontology*: This was the most difficult ontology to create, as it has to be built from *‘de novo’*. An attempt was made to find an existing public ontology that met the needs of preclinical safety pathologists but none were available that matched the terminology currently used. Therefore, eTOX worked closely with pathologists at Novartis and GlaxoSmithKline to create the backbone of a new ontology. This ontology will be used to map all terms existing in the Novartis preclinical safety database (approximately 20,000 terms). It will form the basis for the mapping of all other findings extracted from the preclinical study reports of the different EFPIA partners as described in section 3.3 above.
- *Clinical Chemistry and Toxicology Ontology*: This ontology will be based on the terminology described recently in the CDISC Standard for Exchange of Nonclinical Data (SEND) documentation [36]. This is a relatively simple ontology compared to the other ontologies as there are only about 270 terms in the Novartis database, all of them being relatively unique (i.e., not many synonyms), hence it will be straightforward to map the terms of all companies to this ontology. An advantage of using the SEND terminology consists in the optional automatic mapping of preclinical to clinical data.
- *Cell and Tissue Type Ontology*: Novartis is also working on a cell and tissue type ontology to complement the anatomy ontology that is intended to be shared within the eTOX consortium. The rationale behind separating this ontology from the main anatomy ontology is similar to that for separating findings from organs, namely that the same cell types occur in many different tissues. For example, epithelial cells exist everywhere in the body. Creating a ‘child’ in the anatomy ontology with epithelial cells will link many different terms throughout the body – skin epithelium, lung epithelium, vascular epithelium, *etc.* Managing those links would be almost impossible. Simple keyword searches/text mapping searches in the cell type ontology will automatically provide the links and, since this ontology is rather small, it should not result in a loss of performance. As starting point for developing this ontology, Novartis has taken the ontology maintained by the Jackson Laboratories, which is available from the OBO Foundry [37].
- *Macroscopic and In-Life (“Clinical Findings”) Ontology*: This is another ontology that needs to be built from the beginning which will allow the standardization of macroscopic observational findings, e.g., skin lesions or hair loss observed during preclinical studies. It is a relatively simple ontology, as here too there are only a few hundred, relatively unique, related terms in the Novartis database, making easier the mapping of the terms of all companies to this ontology. Whether the terms used here may also be mapped to the MedDRA terminology [38] is also under investigation.
- *Species and Strain Ontology*: This is a simple ontology that describes different characteristics of the animals used in preclinical safety studies. While it will be based on the complete taxonomy ontology [39], it will consist of simpler associations, such as the term “rodents,” as the parent of different rat and mouse strains, as well as hamster, gerbil *etc.*, to allow for simple grouping of species.
- *Study Design Vocabularies*: They will describe the basic study design parameters, such as time, dosage, route *etc.*, and will consist of preferred terms and synonyms, but will not be hierarchical.

### 3.6. Development of *in Silico* Models for Prediction of Toxicity & Off Target Pharmacology

As described above, currently available *in silico* models cover only a small proportion of the toxicological endpoints relevant for drug discovery and development in pharmaceutical companies as there are huge differences in the prerequisites for their successful development. Basically, difficulties in model development comprise (i) the lack of suitable data for model training and (ii) the complexity of the physiological phenomena involved in the *in vivo* endpoints.

Regarding difficulty (i), the training of predictive models typically requires the availability of a large amount of high quality data, and a substantial series of compounds for which the value of the endpoint has been accurately determined. Ideally, the compounds included in these series should be designed to cover a significant part of the druggable chemical space and the determinations should be made using standardized, reliable experimental methodologies yielding comparable results. In practice, the data available from public sources is far from ideal in practically all these aspects and typically, the series are small and contain highly similar compounds, often representing congeneric series. The aggregation of these series only permits the creation of non-homogeneous datasets in which the experimental results have been obtained with highly diverse experimental procedures, thus being non-comparable and not amenable to statistical analysis. For instance, the data generated using simple procedures (e.g., *in vitro* hERG inhibition) can be used for modeling, since the results (e.g., IC_50_ > *K*_i_) are standardized and comparable between compounds. Nevertheless, data extracted from toxicological reports cannot be compared, for several reasons. First and foremost, the doses are adjusted for each compound in order to obtain visible toxic results; this is done using non-homogeneous criteria (e.g. multiples of expected therapeutic doses, or doses around the value for which toxic effects have been observed in preliminary assays). Moreover, the reports tend to suffer from a “positive bias” in the sense that they particularly record “positive” findings, i.e., deviations from normal values. Likewise, animals from control groups are not devoid of abnormalities, therefore constituting a heterogeneous background of “normal values”.

The difficulty (ii) is related to the complexity of the phenomena that the *in silico* models are intended to predict. In some cases, the toxicological outcome depends on relatively simple properties of the drug candidate. A good example of this is drug-induced phospholipidosis (PL). Even if the mechanism involved in PL is unclear, most of the drugs producing phospholipidosis are cationic amphiphilic drugs (CADs) [40,41]. Therefore, any method able to recognize the presence of a positive charge and a lipophilic moiety in the drug is able to produce reasonably good predictions and, not surprisingly, these models are in the catalogue of models applied in the pharmaceutical industry with good results. However, since not all CADs induce phospholipidosis and some drug inducing PL are not CADs [42,43], even in this case improvements can still be made. Furthermore, there are many other toxicological outcomes, such as hepatotoxicity, that depend on numerous diverse known biological mechanisms [44–49] and probably many more unknown ones. Clearly, no single *in silico* approach can be expected to produce a general description of a mechanistically heterogeneous endpoint comprising various phenotypes, pointing to the need for a more comprehensive approach.

The strategies implemented in eTOX for overcoming the first category of difficulty are intrinsic to the project design. The pharmaceutical industry has generated a large amount of data during the process of drug development, most of which has never been compiled in aggregated electronic formats or exploited in any way. Therefore, the data extracted during the project will be collected and compiled in a general database, in formats allowing its use for building *in silico* predictive models. With respect to the second category of difficulty, the consortium will use standard methodologies for simple endpoints. For complex endpoints, the strategy will be directed to the identification of the simpler (key) mechanisms involved and the derivation of predictive models addressing them specifically. These predictions will then be integrated, taking advantage of our knowledge about the physiological mechanisms involved, for yielding a prediction of the main, observable toxicological outcome. That this theoretical approach will have difficulties for its practical application is acknowledged, however, some proof of concept applications have already been investigated [8]. In the study undertaken by Obiol *et al.*, simulated electrocardiograms were obtained and direct estimations of the induced QT elongation produced by the administration of a compound by integrating *in silico* blocking predictions for two separate ion channels (hERG and KCNQ1) using electrophysiological models that represent the effect of the drug at cell and tissue levels. This kind of approach, much more representative of the complex chain of events leading to cardiotoxicity, produced better prediction for some test compounds than *in vitro* methods based on hERG inhibition only.

In essence, the implementation of the eTOX modeling strategy in practice will require the development of a large collection of single models, each one producing predictions for a relevant toxicological endpoint (for the simplest cases) or a single mechanism involved in a complex toxicological effect, together with an evaluation of pharmacological aspects with diverse validated computational approaches to predict the affinity profile of small molecules against those proteins with a somehow intrinsic role in toxicity events. Special attention to well-known target and off-target behavior will help anticipate those drug side effects caused by exaggerated pharmacology and extend reasoning criteria to complete the toxicological risk assessment for each molecule queried in eTOXsys [50]. The pharmacological analysis will be focused on the set of cytochromes P450, transporters and others targets like nuclear receptors, G protein-coupled receptors, phase II enzymes, kinases, proteases, *etc.* provided by different EFPIA partners as relevant proteins in those toxicological events that the eTOX project aims to predict.

### 3.7. The Integrated Prediction System: eTOXsys

As stated in the introduction, one of the main outcomes of the project will be an *in silico* prediction system, the so-called eTOXsys. The eTOXsys can be described as a software tool able to provide useful toxicological risk and hazard assessment, starting from a simple input that, typically, is limited to the 2D structure of a compound. For the prediction of complex *in vivo* endpoints, which require the calculation of several variables from the structural information (e.g. log D, volume of distribution, absorption, *etc.*), it is intended to provide the possibility to alternatively input these experimental data, if available, to reduce uncertainty in the prediction.

The core of the eTOXsys will be a data mining tool, which will interface the database to deliver nearest neighbors of the compound to be predicted in terms of chemical structure, but also pharmaco-toxicological similarities. In addition, the data mining interface will provide the data set for subsequent model building or evaluation. The tool will contain a task record facility (audit trail). Technical validation aspects laid down in OECD guideline (95)115 will be considered.

The current strategy for the development of predictive tools for organ toxicity from the collected data of systemic toxicity studies is to assign the individual parameters measured in an *in vivo* study to specific organs (e.g. transaminases, bilirubin to liver, troponin to heart, *etc.*). Subsequently, the observed changes of these parameters will be attributed to levels of severity based on the conventional toxicological knowledge and the assessment provided in the original reports. Based on the analyses, data sets will be created from which models for each organ can be constructed. If several models exist for individual organs, the intention is to combine these with a reasoning engine, again built on classical toxicological experience (e.g., if, for “compound x,” “transaminase” is “weakly elevated” and “no histopathological findings in liver” then, “low probability for liver toxicity”).

#### 3.7.1. The Conceptual Design of the eTOXsys

From a technical point of view, eTOXsys will be a unified software platform integrating the various tools, databases and results achieved during the course of the project. This integrated software system will provide access to all existing and developed predictive models and databases through a uniform user interface to support the hazard identification and risk assessment of drug candidates.

To define the requirements of the predictive system, the eTOX partners FIMIM and Molecular Networks carried out surveys to assess both EFPIA partners’ expectations in such a system, as well as the expertise, know-how and skills of the academic organizations and SMEs which will mainly be involved in the development of the predictive models and software.

On the EFPIA side, the results of the survey indicated that the software will be used in the drug discovery & development stages to save money, time and animals and to design new compounds, prioritize compounds and to decide testing strategies. The system may also be applied for the assessment of impurities or synthesis intermediates. A user group consisting of toxicologists, medicinal and computational chemists, pharmacologists and biologists is envisaged. Real use cases will be compiled by means of another questionnaire that will be presented to the consortium members alongside a User Centered Design methodology.

As the modelers are using a diversity of programming languages, software tools and techniques, the results of the surveys suggested the implementation of a distributed and decoupled system architecture based on the web services paradigm. The eTOXsys platform is managed by a lightweight server (eTOXsys server) centralizing the communication between the end user and the components of the system: the prediction models, the eTOX database, other external databases and the reasoning module consolidating the data and the predictions. At present, every module is being hosted by the partner in charge of its development, but in the final version, the whole system will be installed within the company facilities, ensuring that no sensitive information is transferred via the internet. Furthermore, authentication is handled by a web service linking existing company user management systems (such as LDAP or Active Directory) to the eTOXsys. All web service modules will communicate through a well-defined REST Application Programming Interface (API).

**Figure 5:**
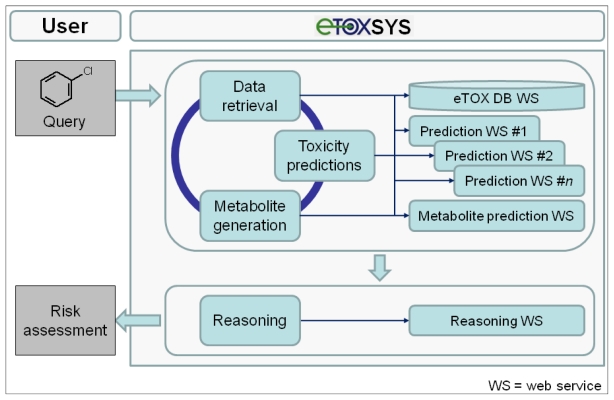
Conceptual design and a potential workflow of the eTOX system (eTOXsys, prediction system established by eTOX).

The conceptual design and potential workflow of the eTOX system are outlined in Figure 5. On the client (user) side, the interface to the eTOX system is run in a standard web browser. In general, the eTOX system is chemical structure-centered and workflows are oriented on operations related to chemical structures and their features and properties, but in a fully flexible and user-defined manner. For an investigation, the user can submit queries to the system by either entering a 2D structure sketch through a graphical molecule editor or by uploading a structure file (e.g., a single or multi-record SDFile). The eTOX database (eTOX DB) is plugged into the system by a web service hosted by Lhasa Limited that provides unified access to the stored data. For structure queries, the eTOX database can be searched in full structure or substructure mode. Furthermore, the system supports text-based searches, such as for registry numbers, names or properties of chemicals. In the final version of the system, the user will optionally also be able to enter measured data with the query, e.g., from *in vitro* experiments, which are then used in the prediction services as these values are likely to be more valid than calculated values. The results from a database query are presented in chemical table and compound views which can be sorted, further refined and adjusted to the needs of the user. Missing data points and information that is required for analyzing the risk potential of a chemical can be predicted by various *in silico* models for toxicity endpoints and ADME parameters (see section 3.7.2 "The predictive models battery") which are registered and available in the system as web services. In addition, the concept of DMPK-related toxicity is taken into account. Potential phase I and II metabolites of the query (parent) compound(s) can be either retrieved from the eTOX database or, if no or limited information is available, generated by a metabolite prediction web service. The retrieved and predicted metabolites can be re-submitted to the above-described database query and toxicity prediction processes. After all necessary and available information has been gathered, a reasoning engine will optionally consolidate the data and predictions obtained from the various services in order to support the user to assess the potential hazard or safety of the queried compound(s). Compound datasets and associated information that have been compiled from search results can be stored, managed and shared among user groups or exported in spreadsheet-compatible formats (e.g., tab or comma-separated value files) for further analysis from which reports can be generated.

In summary, the final eTOXsys will be a Decision Support System collecting the individual predictions of each separate service, evaluating the overall outcome and providing guidance to the user on the basis of the evaluation and consolidation of the data available. At each step, the system will be sufficiently transparent to enable the user to drill down into the result to determine the underlying data and information and methodologies that led to the predictions. However, confidential data of the individual partners will not be disclosed at any stage. For the eTOX database and database web service in particular, encryption, strict authentication and access rights policies and secure communication protocols will be employed to secure proprietary information and data.

The eTOX system will support the inclusion of different types of predictive models including QSAR algorithms, machine learning techniques and knowledge-base expert systems. A mixed license environment will be operated so that proprietary and open source web services can be incorporated for greatest extensibility. However, web service providers will be asked to use open source components whenever possible.

The models available as web services in the eTOX system will mostly incorporate their own algorithms and descriptor generators. However, to avoid unnecessary duplication of computational resources, the integrated system will also support the inclusion of common algorithms and descriptor generators that are frequently used.

The intention is for the models to initially be hosted by each of the model developers and as the project progresses to move towards the models being installed in-house on the user organization’s own server to allow training or validation of models using in-house data.

Dynamic registries of available prediction web services and database services are foreseen. These registries will continuously check the availability of known services and dynamically register newly deployed services. If a service is not available during the status check, the service provider will be notified about the problem.

As there are likely to be differences in the acceptance of particular models by different users, for example on the basis of internal validation exercises, the user will be able to select the models and services to be applied in the queries. The default option will be to run all available and applicable models, but the user will also be able to choose to run just one (e.g., after a model has been upgraded) or only a sub-selection. If no predictions are available, the system will report this.

The advantages of the eTOXsys result from two major achievements initiated and implemented by the eTOX consortium. First, the flexibility of a state-of-the-art web service-based system allows for a seamless integration of various distributed modules and services provided, maintained and further developed by different expert groups as well as the inclusion of company-adopted authentication mechanisms or data access policies. Secondly, the level of quality and originality of the data and information provided by the EFPIA partners enables the computational chemists and toxicologists to better capture real-life challenges with their models and to transfer information into valuable knowledge.

#### 3.7.2. The Predictive Models Battery

In the previous sections, the eTOX strategy for the development of *in silico* prediction methods was advanced. The implementation in practice will require the development of a large collection of single models, each one producing predictions for a relevant toxicological endpoint (for the simplest cases) or a single mechanism involved in the toxicological effect.

In eTOXsys, the *in silico* methods used for deriving the predictions fall into three main categories: (i) pure-chemistry based methods (ii) QSAR models (iii) structure-based or mechanistic methods. Pure-chemistry approaches are based on the recognition of molecular fragments or substructures linked to the presence of toxicity (toxicophores). Methods based on the calculation of molecular properties (e.g., pKa) fall also in this category. In QSAR methods, a training set of compounds of known biological properties is used to train a statistical model, describing the relationship between such biological properties and the compound structure. In the last approach, the structure-based or mechanistic methods, the toxicity of the compounds is predicted based on simulations involving the structure of a biomolecule important for the events leading to the toxic effect (e.g., blocking a bile transporter or the potassium hERG ion channel). The choice of the methods depends mainly of the availability of *a priori* knowledge, the relationship between the presence of fragments and toxicity in pure-chemistry methods, a good training series in QSAR methods, and the identity and three-dimensional structure of a highly relevant biomolecule, in structure-based methods. Clearly, this knowledge is not readily available for all relevant toxicological endpoints, and a large part of the effort of developing eTOXsys will be devoted to the compilation and harmonization of knowledge from public and private sources, as described in previous sections.

In a subsequent step, the results produced by the different models will be integrated to enable higher order predictions. The methods used for this integration are diverse. In cases in which several models produce predictions for the same endpoint, the integration only aims to combine the predictions by obtaining a more accurate consensus result. In other cases, the results of DMPK prediction will determine the expected exposure of the targets to the drug. Even further, metabolic models can predict the presence of a given metabolite for which a full panel of predictions will be carried out in turn. Probably, more sophisticated integration approaches will be required for the complex endpoints. In these cases, the predictions describing individual mechanisms will be integrated using mathematical or logical models reflecting *a priori* knowledge about the physiology of the process. As indicated above, in spite of some success of the previously mentioned proof of concept application [8], this latter case suggests a high degree of difficulty and it is unlikely that the present project will generate this type of sophisticated approach for the prediction of all the relevant *in vivo* toxicological outcomes.

#### 3.7.3. The Prediction Models Web Services

The concept of the prediction engine as a collection of independent models fits well with the software architecture described above. Every model is implemented as an independent web service, receiving a well-defined input (typically, the structure of the candidate compound) and yielding an output consisting of a prediction value, together with some additional information necessary for the correct integration of the results (e.g., some scoring of the prediction quality). Moreover, this architecture allows the distributed development of every prediction model, which can be performed in parallel by diverse partners and can be easily extended in the future by the pharmaceutical companies and/or other third parties.

The integration of every single prediction, as depicted in the eTOXsys conceptual design, is carried out by a separate module, also working as a web service, producing an output that will be processed in order to present the results in understandable format. This latter step is also very important, since the end users need to obtain a clear picture of the prediction results, translated in terms of toxicity risks. Different approaches have been published for this task in the field of toxicology [51–54]. In eTOXsys, it is planned to carry out systematic tests to choose the most suitable method or combination of methods. An important factor in the choice of integrative tools is the ability of the method to provide reliability indices and the ability to mine for detailed information on the background of any particular prediction (e.g., the method used, the details of the computation, structure of the nearest compound in the training series, *etc.*).

## 4. Current Achievements and Future Deliverables

The achievements obtained so far in eTOX can be classified in three domains: data gathering and collection, database building, and development of the predictive system.

The data gathering and collection, as a result of the aforementioned need to retrieve and release proprietary data, is progressing at a low pace, slower than envisaged. However, having now completed the pilot study to assess data extraction by potential subcontractors, the project is now in position to move forward rapidly. Also in this domain, the protection of data considered sensitive by their owners and the potential usefulness of diverse state-of-the-art structure masking methods have been tested, analyzing their strengths, weaknesses and potential threats. From this study, it was concluded in agreement with other authors that no structure masking method on its own would provide the level of protection required. However, the particular set-up of the project and the participation of a trusted partner will allow the implementation of original structure protection protocols suitable for the purposes of the project.

Regarding the database building, a first version of the eTOX database using the schema and database infrastructure (Vitic Nexus) described in previous sections is fully operative, and is being fed with real data extracted from the legacy reports. Also, a significant amount of publically available toxicological data has been collected in the ChOX database, which at present contains information for more than 153,520 single compounds. Plans to merge the contents providing a single access point to all the data collected in the project are already in progress. This integrated database will also be accessible as a web service, in accordance to the designed system architecture; a first version of this is undergoing testing.

**Figure 6:**
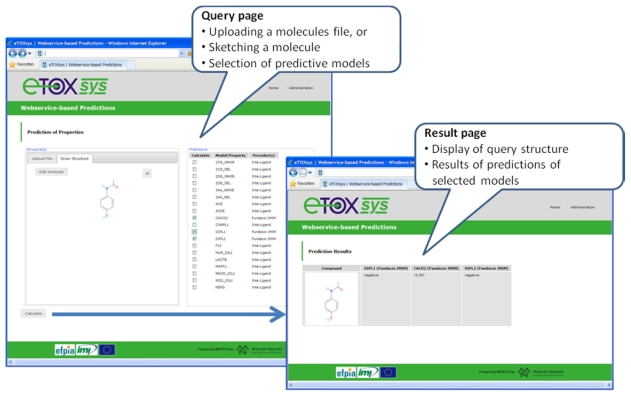
Screenshots of the current version of the eTOX system (eTOXsys).

With respect to the eTOXsys platform, the overall architecture and the communication protocols have been defined (see section 3.7). Several of the modules configuring the system have been developed and are provided as web services independently at the sites of the respective developing partners. A pilot study, focused on the prediction of a simple *in vivo* endpoint (phospholipidosis), was run among the partners with positive results. A first proof of concept (POC) version of the eTOXsys has been implemented and presented to the consortium to demonstrate the interplay of user interface and predictive models provided as web services. Figure 6 shows a screenshot of this POC version. A first prototype of the eTOXsys for internal review by the consortium is planned for delivery by end of February 2012.

Additionally, a great deal of effort has been put into building the project intranet, which constitutes a portal to numerous and valuable resources, including an updated index of toxicological bibliography, links to diverse toxicology databases and public datasets. The portal also hosts collaborative tools (wiki, discussion forums, *etc.*) and a central repository of management documents.

## 5. Discussion and Outlook

For the first time in pharmaco-toxicological research, a wealth of unused or poorly used, highly relevant preclinical drug safety data will be combined to improve the quality of drug candidates and the processes for their development leading to better safety, a faster process to the benefit of patients and a reduction of animal use.

The database arising from the project is likely to be the largest repository for high quality repeat dose toxicity data currently existing. The number of entries in such a database is obviously important for accurate drug side effects prediction. However, the quality and the chemical space coverage is equally, if not more important. The pharmaceutical toxicity data that will be used have been produced with compounds that have become drugs and many more chemicals that have failed to reach the market. These compounds may have been dropped from further development for many reasons, including safety issues. No matter what, these molecules cover a large chemical space thought to be the best fit for druggable structures. Hence, the nature of the toxicity data collected for the eTOX database represent the best possible space coverage for toxicity prediction of future potential drugs. The relevance of these data is reinforced by the high quality required for GLP studies. Furthermore, existing relevant databases for eTOX, as well as collated, published data, complements in many ways the EFPIA data by increasing the chemical space and the parameters of the eTOX database. These two distinct aspects, relevance of the covered chemical space and data quality, bring together the essential background for accurate *in silico* toxicity prediction.

The computerized software that is being developed in the eTOX project (eTOXsys) will take advantage of these data. Meanwhile, many aspects have to be taken into account for accurate predictions of toxicity. Toxicological events are very complex phenomena which depend on the chemical structure of the drug, the biological targets, their location within the cells, the perturbation of the biological pathways that include the targets, the function of these targets in different organs, the physiology of the whole organism, the pharmacokinetic characteristics of the compound, the potential toxic metabolites, the route of administration, the dose, the dose regimen, the duration of the treatment and the recipient species, all of which play a role which, at present, is far from being well understood and characterized. Therefore, the *in silico* predictions will be based on both relevant knowledge and observations of the overall outcome to animal treatment as described in the toxicity legacy reports. The synergy between the expert knowledge from the pharmaceutical toxicologists and professional input from academic and SME modelers will be critical to the success of the project.

At present, the pharmaceutical industry partners are gathering their proprietary data to be used in eTOXsys and, as mentioned before, thorough security measures have been taken to protect intellectual property rights and secrecy of sensitive data, while still allowing their usage in the training of the predictive system. This specific feature establishes a major difference between eTOX and any open platform like the OpenTox project [18]. Once authorized to be sent out to the consortium, toxicity report data will be accurately extracted, capturing heterogeneous data, acquiring data in a coherent manner from highly different formats from various companies, utilizing the standards and harmonization of procedures already developed. Likewise, to be able to exploit the extracted data, a standardization of terminology is necessary and hence much time and resource has been spent in the development of an accurate ontology for mapping all used terms, from the straightforward description of the study design to complex histopathology findings. Academic partners are also gathering and capturing data and are experiencing difficulties inherent to published data where a quality judgment has to be made. Re-using existing databases for the sake of eTOX also requires careful manipulation of structure and format to prevent compatibility issues.

Overall, the eTOX database is being populated with preclinical toxicology data, mainly from rodents, as well as conventional non-rodent species: dog and more occasionally, monkey. The initial studies collected are classical GLP repeat dose studies and drug metabolism and pharmacokinetics studies in the same species. However, the *in vitro* pharmacology data are mostly from human receptors and targets. There will be necessary additional work to allow translation from pre-clinical to clinical prediction. Furthermore, more complex studies such as reprotoxicity and carcinogenicity reports could, and should, also be included in eTOX. For all these reasons, together with the fact that pharmaceutical companies are constantly generating new toxicity reports, the possibility of extending the scope and duration of this project is being envisaged. In any case, the eTOX database will not be a frozen system at the end of the consortium. Maintenance and improvement of prediction systems, as well as the permanent incorporation of new data, will have to be accommodated.

Within three years from now, the eTOX project and with it, the database and the exploitation systems (both toxicity prediction and data mining), will be close to its scheduled end. At this point it will be clear how many of the identified hurdles will have been overcome and whether or not this consortium will have delivered its promises. However, one battle is already won: pulling together highly competent forces to move forward drug discovery and development to its best possible outcome is already in place and moving forward. Indeed, more than three years ago now, when this project was being conceived, bringing together competing pharmaceutical companies, with academic partners usually more focused on pure sciences than in concrete applications, together with SMEs whose normal function is as providers rather than partners, under a European institution (IMI), was perhaps the biggest challenge of all. This challenge has been taken up by the eTOX consortium with one common goal: improving medicine quality.

## Figures and Tables

**Table 1:** Partners in the eTOX project.

Private	Public	
EFPIA Companies	Academic Institutions	SMEs
**Novartis Pharma**	**Fundació Institut Mar d’Investigacions Mèdiques, Barcelona, Spain**	Lhasa Limited, Leeds, UK
AstraZeneca	Fundación Centro Nacional de Investigaciones Oncológicas Carlos III, Madrid, Spain	Inte:Ligand GmbH, Vienna, Austria
Boehringer Ingelheim	European Molecular Biology Laboratory (European Bioinformatics Institute), UK	Molecular Networks GmbH, Erlangen, Germany
**Bayer HealthCare**	Liverpool John Moores University, Liverpool, UK	Chemotargets SL, Barcelona, Spain
Laboratorios del DrEsteve	Technical University of Denmark, Kopenhagen, Denmark	Lead Molecular Design SL, Sant Cugat del Vallès, Spain
GlaxoSmithKline	Universität Wien, Vienna, Austria	
Janssen Pharmaceutical	Vrije Universiteit Amsterdam, The Netherlands	
UCB Pharma		
H. Lundbeck		
Pfizer Ltd.		
F. Hoffmann-La Roche		
Sanofi *		
Les Laboratoires Servier *		

Organizations leading the project are depicted in bold,

* Companies that joined eTOX after its inception.

Note: Sanofi, formerly Sanofi-Aventis.
